# Supplementary material for: P-TRAP: a Panicle Trait Phenotyping tool
Source: BMC Plant Biol. 2013 Aug 29;13:122. doi: 10.1186/1471-2229-13-122 (PMC3848748; doi:10.1186/1471-2229-13-122)
Supplement: Additional file 7 — P-TRAP source code. The Netbeans project that contains the source code of the application. [file 1471-2229-13-122-S7.zip › javadoc.zip!/javadoc/org-bioalg-graphprocessor/org/bioalg/graphprocessor/BranchModel.html]

BranchModel (GraphProcessor)


JavaScript is disabled on your browser.


- Package
- Class
- Tree
- Deprecated
- Index
- Help

*org.bioalg.graphprocessor 1.0*

- Prev Class
- Next Class

- Frames
- No Frames

- All Classes

- Summary:
- Nested |
- Field |
- Constr |
- Method

- Detail:
- Field |
- Constr |
- Method


org.bioalg.graphprocessor

## Class BranchModel

- java.lang.Object
- - org.bioalg.graphprocessor.BranchModel

- ---

    

  ```
  public class BranchModel
  extends java.lang.Object
  ```

  A general class for representing branches. A branch can be a sub-graph, or at
  least a single edge. They all all managed via paths.

  Author:
  :   faroq

- - ### Constructor Summary

    Constructors

    Constructor and Description

    `BranchModel()`

    `BranchModel(Vertex startVertex)`

    `BranchModel(Vertex startVertex,
    Vertex endVertex)`

    `BranchModel(Vertex startVertex,
    Vertex endVertex,
    GraphModel gm)`
  - ### Method Summary

    Methods

    | Modifier and Type | Method and Description |
    | --- | --- |
    | `boolean` | `calcpath()` calculates path of the branch in the graph |
    | `Path` | `calcStartandEndVertices(Vertex seed)` calculate the start and end vertices, . |
    | `java.util.ArrayList<BranchModel>` | `getChildren(boolean generate)` Return all sub branches of the branch |
    | `Vertex` | `getEndVertex()` |
    | `double` | `getLength()` |
    | `java.util.ArrayList<Path>` | `getSecondaryBranches(Path path)` |
    | `java.util.ArrayList<Path>` | `getStart2endPath()` |
    | `Vertex` | `getStartVertex()` |
    | `java.util.ArrayList<Path>` | `getTertiaryBranches(Path path)` getThe third level branches. |
    | `void` | `setEndVertex(Vertex endVertex)` |
    | `void` | `setStart2endPath(java.util.ArrayList<Path> start2endPath)` |
    | `void` | `setStartVertex(Vertex startVertex)` |

    - ### Methods inherited from class java.lang.Object

      `clone, equals, finalize, getClass, hashCode, notify, notifyAll, toString, wait, wait, wait`

- - ### Constructor Detail


    - #### BranchModel

      ```
      public BranchModel(Vertex startVertex,
                 Vertex endVertex)
      ```


    - #### BranchModel

      ```
      public BranchModel(Vertex startVertex,
                 Vertex endVertex,
                 GraphModel gm)
      ```


    - #### BranchModel

      ```
      public BranchModel(Vertex startVertex)
      ```


    - #### BranchModel

      ```
      public BranchModel()
      ```
  - ### Method Detail


    - #### getEndVertex

      ```
      public Vertex getEndVertex()
      ```


    - #### setEndVertex

      ```
      public void setEndVertex(Vertex endVertex)
      ```


    - #### getStartVertex

      ```
      public Vertex getStartVertex()
      ```


    - #### setStartVertex

      ```
      public void setStartVertex(Vertex startVertex)
      ```


    - #### getStart2endPath

      ```
      public java.util.ArrayList<Path> getStart2endPath()
      ```


    - #### setStart2endPath

      ```
      public void setStart2endPath(java.util.ArrayList<Path> start2endPath)
      ```


    - #### calcpath

      ```
      public boolean calcpath()
      ```

      calculates path of the branch in the graph

      Returns:


    - #### calcStartandEndVertices

      ```
      public Path calcStartandEndVertices(Vertex seed)
      ```

      calculate the start and end vertices, . This function will also generate
      a path if is not exist and will overwrite if exists.

      Returns:
      :   true if success, false otherwise


    - #### getLength

      ```
      public double getLength()
      ```


    - #### getChildren

      ```
      public java.util.ArrayList<BranchModel> getChildren(boolean generate)
      ```

      Return all sub branches of the branch

      Parameters:
      :   `generate` - boolean if true it will follow the branches to generate
          their paths otherwise only start vertex is generated

      Returns:


    - #### getSecondaryBranches

      ```
      public java.util.ArrayList<Path> getSecondaryBranches(Path path)
      ```


    - #### getTertiaryBranches

      ```
      public java.util.ArrayList<Path> getTertiaryBranches(Path path)
      ```

      getThe third level branches.

      Parameters:
      :   `path` - the given path to search in.

      Returns:


- Package
- Class
- Tree
- Deprecated
- Index
- Help

*org.bioalg.graphprocessor 1.0*

- Prev Class
- Next Class

- Frames
- No Frames

- All Classes

- Summary:
- Nested |
- Field |
- Constr |
- Method

- Detail:
- Field |
- Constr |
- Method
